# Relationship between cerebral arterial inflow and venous outflow during dynamic supine exercise

**DOI:** 10.14814/phy2.13292

**Published:** 2017-06-29

**Authors:** Kohei Sato, Naoko Oba, Takuro Washio, Hiroyuki Sasaki, Anna Oue, Aki Otsuki, Tomoko Sadamoto, Shigehiko Ogoh

**Affiliations:** ^1^ Research Institute of Physical FitnessJapan Women's College of Physical EducationTokyoJapan; ^2^ Department of Biomedical EngineeringToyo UniversitySaitamaJapan; ^3^ Department of Food and Nutrition ScienceToyo UniversityGunmaJapan

**Keywords:** Cerebral blood flow, dynamic exercise, internal jugular vein, vertebral vein

## Abstract

The regulation of cerebral venous outflow during exercise has not been studied systematically. To identify relations between cerebral arterial inflow and venous outflow, we assessed the blood flow (BF) of the cerebral arteries (internal carotid artery: ICA and vertebral artery: VA) and veins (internal jugular vein: IJV and vertebral vein: VV) during dynamic exercise using ultrasonography. Nine subjects performed a cycling exercise in supine position at a light and moderate workload. Similar to the ICA BF, the IJV BF increased from baseline during light exercise (*P *<* *0.05). However, the IJV BF decreased below baseline levels during moderate exercise, whereas the ICA BF returned near resting levels. In contrast, BF of the VA and VV increased with the workload (*P *<* *0.05). The change in the ICA or VA BF from baseline to exercise was significantly correlated with the change in the IJV (*r* = 0.73, *P *=* *0.001) or VV BF (*r* = 0.52, *P *=* *0.028), respectively. These findings suggest that dynamic supine exercise modifies the cerebral venous outflow, and there is coupling between regulations of arterial inflow and venous outflow in both anterior and posterior cerebral circulation. However, it remains unclear whether changes in cerebral venous outflow influence on the regulation of cerebral arterial inflow during exercise.

## Introduction

The precise regulation of cerebral blood flow (CBF) is critical for the maintenance of constant nutrient and oxygen supply to the brain during exercise (Ide and Secher 2000; Secher et al. 2008; Ogoh and Ainslie 2009), because of the limitation of capacity in the brain tissue for substrate storage or high metabolic rates. Thus, to maintain exercise performance, the change in CBF is also of critical importance (Nybo and Nielsen 2001; Trangmar et al. 2015). Under this background, many studies have investigated CBF regulation during exercise (Nybo and Nielsen 2001; Ogoh and Ainslie 2009; Sato and Sadamoto 2010; Sato et al. 2011; Trangmar et al. 2015).

We observed a heterogeneous CBF response to semi‐supine exercise (Sato and Sadamoto 2010; Sato et al. 2011). For example, the vertebral artery (VA) BF (i.e., the posterior CBF) continuously increases during an incremental exercise workload, although the internal carotid artery (ICA) BF (i.e., the anterior CBF) decreases at a higher intensity (Sato and Sadamoto 2010; Sato et al. 2011). However, the mechanism of different BF responses to exercise between these cerebral arteries remains unknown. Different cerebral CO_2_ reactivity and dynamic autoregulation between the cerebral arteries and the increase in the external carotid artery (ECA) BF for thermoregulation may contribute to the heterogeneous CBF response (Sato and Sadamoto 2010; Sato et al. 2011, 2012; Bain et al. 2013), but these factors alone cannot explain these CBF responses.

Regarding the cerebral circulation, Intra‐ and extracranial veins are considered capacitance vessels, and cerebral venous outflow is regulated by a Starling resistor model or vascular waterfall phenomenon (Valdueza et al. 2000; Gisolf et al. 2004). Therefore, cerebral venous outflow should be related to the arterial inflow. The internal jugular veins (IJV) seems to be the main route of venous outflow (Valdueza et al. 2000; Gisolf et al. 2004; Chung et al. 2010; Gadda et al. 2015) because IJV blood flow (∼700 mL/min; Schreiber et al. 1985; Valdueza et al. 2000; Doepp et al. 2004; Chung et al. 2010) is similar with total arterial CBF of ∼750 mL/min (Scheel et al. 2000). An extra‐jugular system consisting of the vertebral veins (VV) and deep cervical veins is also thought to be important cerebral venous drainage (Schreiber et al. 1985; Valdueza et al. 2000; Doepp et al. 2004). In addition, the external jugular veins (EJV) receive larger amounts of blood from the exterior of the cranium and the deep parts of the face (i.e., the extracranial circulation). When IJV blood flow was restricted, extra‐jugular system should work to maintain venous drainage in cerebral circulation (Schreiber et al. 1985; Valdueza et al. 2000; Dawson et al. 2004; Doepp et al. 2004; Gisolf et al. 2004).

In the previous investigations, the effect of cerebral venous outflow on CBF has been reported (Bayliss et al. 1895; Shinohara et al. 1982; Ivanov et al. 2013; Tsao et al. 2014; Thibault et al. 2015). Wilson et al. (2015) demonstrated that active cerebral venoconstriction contributed to changes in the brain blood volume during sympathoexcitation. In clinical studies, chronic cerebral venous insufficiency was associated with impaired cerebral perfusion (Shinohara et al. 1982; Thibault et al. 2015). Moreover, abnormal IJV flow patterns impair cerebral venous drainage and cause consequent CBF reduction (Tsao et al. 2014). During upright exercise, the abnormal IJV due to marked negative IJV pressure in presyncopal patients, which then impairs adequate CBF regulation (Olesen et al. 2014). These findings provide the possibility that change in cerebral venous outflow modifies CBF regulation. More recently, indeed, we observed that the VA BF changes were highly associated with the VV BF changes during CO_2_ manipulation in both supine and upright positions (Ogoh et al. 2016). However, the response of cerebral venous outflow to dynamic exercise remains unknown.

Therefore, we hypothesized that dynamic exercise modifies the BF distribution of cerebral arteries, and this modification potentially contributes to the regulation of cerebral venous outflow. To identify relations between CBF and cerebral venous outflow, we assessed BF at the head and cerebral arteries and veins during submaximal supine exercise using ultrasonography. In this study, we used a supine cycle exercise, because the IJV collapses in the upright position (Valdueza et al. 2000; Dawson et al. 2004; Gisolf et al. 2004; Ogoh et al. 2016), thus blood flows to an alternative venous pathway (i.e., the VV).

## Methods

Nine healthy subjects (five men and four women, age 21.7 ± 0.2 years, height 164.4 ± 2.7 cm, weight 61.6 ± 3.3 kg) participated in this study. They were all normotensive without any cardiovascular, pulmonary, or kidney disease. Smoking and the consumption of caffeine and alcohol were prohibited during the study period. The study protocol was approved by the Ethical Committee for Human Research at Japan Women's College of Physical Education. Each subject provided written informed consent to participate in the study. The study was also conducted in accordance with the principles of the Declaration of Helsinki.

### Experimental protocol

On the day of the experiment, subjects arrived at the laboratory at least 2 h after a light meal. After instrumentation was performed, the subjects rested quietly for 30 min in supine position. The protocol consisted of baseline (BL) (supine rest), followed by a submaximal cycling exercise at two workloads. After the BL measurement, each subject performed a light (EX1) cycling exercise in supine position. Subjects began cycling at 20 W (2 min), which was subsequently adjusted to achieve the target heart rate (HR) of 100–110 beats/min. After confirming that the cardiorespiratory variables were in a steady state, the BFs were measured. In this study, BFs in the neck conduit arteries (ECA, ICA, and VA) and veins (IJV, VV, and EJV) were randomly measured for 45 s in each vessel, and the time required to measure all the BFs was recorded; in most cases, each exercise stage lasted 10 min. After all BF measurements were obtained, the workload was increased to achieve the next target HR of 150–160 beats/min (EX2); thereafter, the final BF measurement was recorded. Judging from our previous studies (Sato and Sadamoto 2010; Sato et al. 2011), EX1 corresponds to ∼40% of peak oxygen uptake (VO_2 peak_), and EX2 corresponds to ∼70% VO_2 peak_.

### Blood flow measurements

#### Arteries

BFs in the right ICA and right ECA were measured 1.0–1.5 cm distal to the carotid bifurcation, and the right VA BF was measured at the midpoint of the V1 segment.

#### Veins

**Figure 1:**
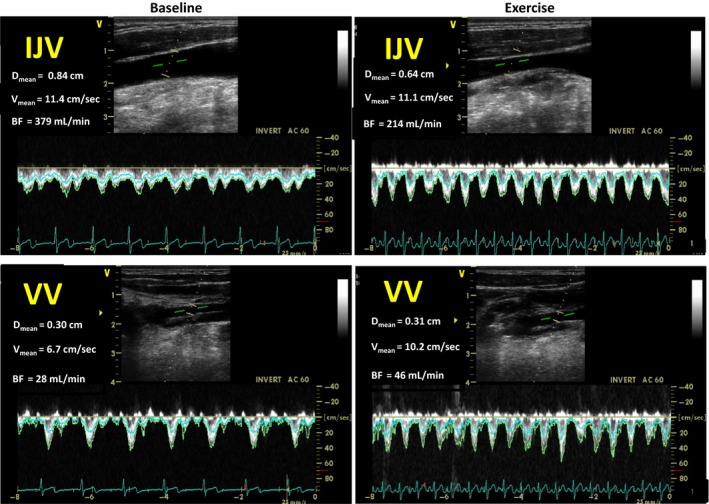
Doppler ultrasound images showing the internal jugular vein (IJV, upper panels) and vertebral vein (VV, lower panels) with Doppler flow waveform during baseline (left panels) and moderate exercise (right panels).

The BF was measured at the J3 level of the right IJV (i.e., as distally as possible in the IJV before it passes through the jugular foramen into the skull) and at the mid cervical level of the right VV (Fig. 1), and at most visible segment right EJV on skin surface of the neck.

#### Blood flow velocity

For all BF measurements, the time‐averaged mean flow velocity (V_mean_) was obtained by pulsed wave mode in a longitudinal plane with special care taken to avoid vein compression. V_mean_ was obtained from the average of 15 sec to eliminate the effects caused by the breathing cycle. When obtaining the V_mean_ measurement, care was taken to ensure that the probe position was stable, insonation angle did not vary (60°), and sample volume was positioned in the center of the vessel.

#### Vessel diameter

We used the brightness mode to measure the vessel diameter of each vessel in the longitudinal aspect. For the arteries, the systolic and diastolic diameters were measured carefully over several cardiac cycles (five cycles) and averaged. Then the mean diameter (D_mean_) was calculated as follows: arterial D_mean_ = [(systolic diameter × 1/3)] + [(diastolic diameter × 2/3)]. On the other hand, the diameter of the cerebral veins depends on both the cardiac and respiratory cycles (it decreases in diastolic blood pressure and inspiration, and increases in systolic blood pressure and expiration). Therefore, the maximum and minimum vein diameters were calculated over 10 cardiac cycles to account for the oscillatory effects caused by respiration or swings in intra thoracic pressure and then averaged. However, a calculation formula for the D_mean_ that considers the cardiac cycle as that for the arteries above has not been established for the veins; thus, we calculated the D_mean_ using the following formula: vein D_mean_ = (maximum diameter + minimum diameter) ÷ 2.

#### Blood flow

Finally, BF was calculated by multiplying the vessel cross‐sectional area, [*π*× (mean diameter ÷ 2)^2^], with the V_mean_: BF (mL/min) = V_mean_ × cross‐sectional area × 60.

The coefficient of variation (CV) results from repeated venous BF measurements in pilot study at rest were IJV: 5.6 ± 3.5% (mean ± SD) and VV: 5.3 ± 3.7%, while those during moderate exercise were IJV: 5.7 ± 4.0% and VV: 5.7 ± 4.1%. In this study, we calculated the cross‐sectional area using the length of minor axis of the vessel in the longitudinal aspect, which assume that vein is circular, while in fact, it is more elliptical in shape (Ogoh et al. 2016). To identify the validity of our method for calculating a cross‐sectional area of the IJV, we examined the relationship between IJV cross‐sectional area calculated using the diameter of the vessel and the actual cross‐section in 15 subjects. There was a highly significant relationship between cross‐sectional values between the two methods (*r* = 0.98, *P *<* *0.001).

### Cardiorespiratory measurements

Expired air was sampled breath‐by‐breath, and end‐tidal partial pressure of carbon dioxide (P_ET_CO_2_) was measured with a gas analyzer system (AE300S; Minato Medical Science, Tokyo, Japan). The HR was monitored continuously using a three‐lead electrocardiograph (Radercirc; Dainippon Sumitomo Pharmacology, Tokyo, Japan). The systolic and diastolic arterial pressures were measured using a cuff sphygmomanometer (Radercirc; Dainippon Sumitomo Pharmacology), and the mean arterial pressure (MAP) was calculated as follows: [(2 ×  diastolic pressure) + systolic pressure] ÷ 3.

### Statistical analysis

Values are expressed as means ± standard deviations. Changes in the BF and cardiorespiratory variables during light and moderate exercise were compared by one‐way repeated‐measures analysis of variance with Bonferroni's post hoc test. Pearson's product moment was used to perform correlation analyses. SPSS, version 19.0 (IBM Corp., Tokyo, Japan) was used to perform the statistical analyses. The level of significance was set at *P *<* *0.05.

## Results

**Figure 2:**
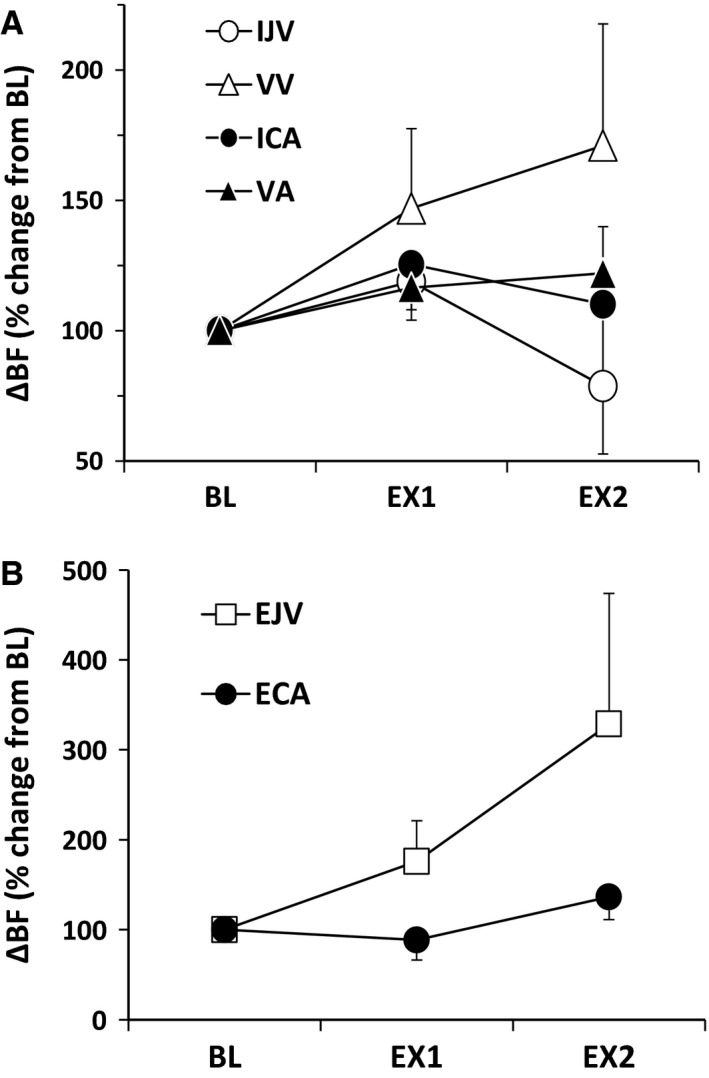
Relative change (%) in cerebral (*A*) and head (*B*) arterial inflow and venous outflow during supine exercise. BL, baseline; EX1, light exercise; EX2, moderate exercise; ICA, internal carotid artery; VA, vertebral artery; IJV, internal jugular vein; VV, vertebral vein; ECA, external carotid artery; EJV external jugular venous; BF, blood flow.

The absolute (mL/min) and relative changes (%) in cerebral venous and arterial blood flows during dynamic exercise are shown in Table 1 and Figure 2, respectively. Both the ICA BF and VA BF increased significantly from BL to EX1 (ICA 25 ± 17% and VA 16 ± 12%, *P *<* *0.05). Thereafter, the ICA BF decreased near the BL value at EX2 (10 ± 27%, *P *=* *1.00), whereas the VA BF was well maintained even at EX2 compared with BL (22 ± 18%, *P *=* *0.02). The ECA BF slightly decreased at EX1 compared with BL (‐13 ± 22%, *P *>* *0.05) and then it largely increased at EX2 compared with BL and EX1 (36 ± 25%, *P *<* *0.01). Similarly to change in the ICA BF, the IJV BF increased from BL to EX1 (19 ± 15%, *P *=* *0.035), and yet the IJV BF decreased under the BL value during EX2 compared with EX1 (−21 ± 26%, *P *=* *0.008). The decrease in the IJV BF was attributable to the decreased mean vessel diameter at EX2 compared with EX1 (*P *=* *0.024). Both the VV and EJV BFs progressively increased from BL to EX2 (VV 71 ± 47% and EJV 229 ± 145%, *P *<* *0.05), with an increase in the mean BF velocity (*P *<* *0.05). The P_ET_CO_2_ increased during EX1; however, it was significantly lower during EX2 than during EX1 (*P *=* *0.033). The MAP significantly increased from BL to EX2 (*P *=* *0.001), but not at EX1. The HR significantly increased with workload (*P *=* *0.001).

**Table 1:** Cerebral arterial and venous blood flows, and cardiorespiratory variables at baseline and during light and moderate supine exercise

	BL	EX1	EX2
Arterial inflow			
Internal carotid artery blood flow, mL/min	316 ± 77	391 ± 85^a^	345 ± 122
Vertebral artery blood flow, mL/min	87 ± 32	101 ± 37^a^	107 ± 40^a^
External carotid artery blood flow, mL/min	224 ± 61	187 ± 42	296 ± 69^a^, ^b^
Venous outflow			
Internal jugular vein blood flow, mL/min	413 ± 162	498 ± 220^a^	334 ± 192^b^
Mean diameter, cm	0.66 ± 0.14	0.67 ± 0.13	0.60 ± 0.11^a^, ^b^
Mean blood flow velocity, cm/s	20.8 ± 6.8	23.0 ± 6.5	18.6 ± 7.5
Vertebral vein blood flow, mL/min	70 ± 61	96 ± 68^a^	120 ± 111^a^
Mean diameter, cm	0.31 ± 0.09	0.32 ± 0.11	0.32 ± 0.10
Mean blood flow velocity, cm/s	14.0 ± 5.1	18.8 ± 7.1	21.0 ± 6.3^a^
External jugular vein blood flow, mL/min	27 ± 14	50 ± 28	94 ± 67^a^
Mean diameter, cm	0.25 ± 0.05	0.25 ± 0.07	0.27 ± 0.07
Mean blood flow velocity, cm/s	9.2 ± 3	15.6 ± 5.5^a^	23.5 ± 7.2^a^, ^b^
Cardiorespiratory variables			
petCoj, mmHg	39.7 ± 0.9	44.5 ± 0.8^a^	42.1 ± 1.0^a^, ^b^
Heart rate, beats/min	62 ± 4	102 ± 2^a^	155 ± 2^a^, ^b^
Mean arterial pressure, mmHg	82 ± 2	93 ± 5	98 ± 2^a^, ^b^

BL, baseline; EX1, light exercise; EX2, moderate exercise; P_ET_CO_2_, end‐tidal partial pressure of carbon dioxide.

a Compared with BL (*P *<* *0.05)

b Compared with EX1 (*P *<* *0.05). Data are presented as a mean ± standard deviation.

**Figure 3:**
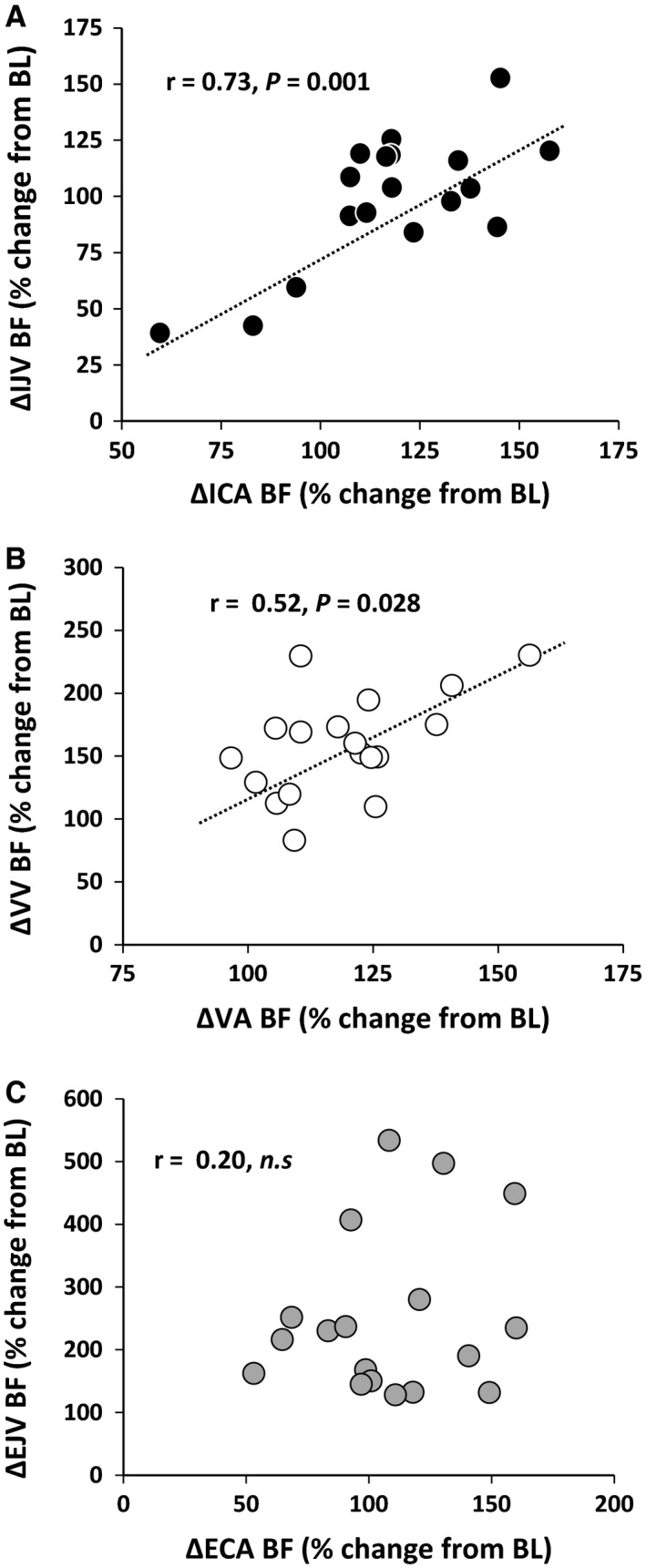
Relationship between the change (%) in the ICA BF and IJV BF (*A*), VA BF and VV BF (*B*), and ECA BF and EJV BF (*C*) during exercise. BL, baseline; EX1, light exercise; EX2, moderate exercise; ICA, internal carotid artery; VA, vertebral artery; IJV, internal jugular vein; VV, vertebral vein; ECA, external carotid artery; EJV, external jugular vein; BF, blood flow.

The relative change (%) in the ICA or VA BF from BL to exercise was significantly correlated with the change in the IJV (*r* = 0.73, *P *=* *0.001, Fig. 3A) or VV BF (*r* = 0.52, *P *=* *0.028, Fig. 3B), respectively. However, there was no significant correlation between the ECA and EJV BF (*r* = 0.20, *P *>* *0.05, Fig. 3C). The change in the IJV BF from EX1 to EX2 was not significantly correlated with the change in the VV BF (*r* = −0.50, *P *=* *0.172).

## Discussion

The main findings of this study were that supine exercise‐induced change in ICA or VA BF is related to that of IJV or VV BF, respectively. These findings suggest that dynamic supine exercise modifies the cerebral venous outflow, and there is coupling between regulations of arterial inflow and venous outflow in both anterior and posterior cerebral circulation.

It is well known that cerebral venous outflow is regulated by a Starling resistor model or vascular waterfall phenomenon (Valdueza et al. 2000; Gisolf et al. 2004), and thus cerebral arterial inflow would be related to the venous outlow in the brain to maintain the balance between arterial and venous blood volume. As expected, our results suggest that dynamic supine exercise modifies the BF in the cerebral veins. Importantly, we observed a high correlation between the changes in ICA and IJV BF (*r* = 0.73, *P *=* *0.001, Fig. 3A), which might be because the right IJV is normally representative of the drainage from the cortical area supplied by the ICA during exercise (Himwich et al. 1947; Suzuki et al. 2001; Chung et al. 2010). In addition, changes in the VV BF correlated with changes in the VA BF (*r* = 0.52, *P *=* *0.028, Fig. 3B), which potentially suggests that the VV is the main outflow pathway from the posterior cerebral portion (Ogoh et al. 2016). In contrast to cerebral circulation, there was no correlation between the ECA and EJV BF during exercise (*r* = 0.20, *P *>* *0.05, Fig. 3C). The increase in the EJV BF accompanied by the increase in the ECA BF was associated with thermoregulation during exercise (Sato et al. 2011; Bain et al. 2013). These observations support the idea that an additional anastomotic pathway carries part of the extracranial BF to the IJV at J1 segment (i.e., a facial vein) (Gadda et al. 2015).

Schreiber et al. (1985) demonstrated that IJV compression bilaterally resulted in an increase in the VV BF. This finding suggests that the anterior BF transmit to the posterior venous outflow with the limit of IJV flow. Similarly, in thisstudy, the VV BF increased with decrease in IJV BF during moderate exercise. However, the change in the IJV BF from light to moderate exercise was not correlated with the change in the VV BF (*r* = −0.50, *P *=* *0.172). Also, the magnitude of the increase in the VV BF (+20 mL/min, Table 1) from light to moderate exercise did not correspond with the decrease in the IJV BF (−160 mL/min). Therefore, there is not an interaction in regulations of arterial inflow and venous outflow between anterior and posterior cerebral circulation during dynamic exercise.

Similar to that in previous studies (Sato and Sadamoto 2010; Sato et al. 2011), the ICA BF decreased near the BL during moderate exercise, whereas the increase in the VA BF was well maintained. The mechanism of the decrease in ICA BF during exercise remains unclear, but it appears to be partly due to hyperventilation‐induced hypocapnia during dynamic exercise and/or sympathoexitation (Ide and Secher 2000; Secher et al. 2008; Ogoh and Ainslie 2009). The different BF responses between the ICA and VA may be due to differences in the regional metabolic demand and cerebral CO_2_ reactivity to dynamic exercise (Sato and Sadamoto 2010; Sato et al. 2011, 2012). Moreover, aside from such global factors, other local hemodynamic factors may also contribute to this difference. Importantly, there was a positive relationship between the change in the IJV BF and the change in ICA BF during exercise. Some clinical studies demonstrated the effect of venous drainage on CBF in the patient with cerebral disease (Shinohara et al. 1982; Ivanov et al. 2013; Thibault et al. 2015). These findings provide the possibility that the regulation of the IJV BF partly contributes to a change in the ICA BF. The regulation of IJV BF is also associated with cerebral blood volume control which alters cerebral hemodynamics via changes in intracranial pressure (Shinohara et al. 1982; Thibault et al. 2015). Indeed, it has been reported in human study that an obstruction of IJV alters arterial cerebral BF (Shinohara et al. 1982). However, it remains unclear whether changes in cerebral venous outflow influence on the regulation of CBF during exercise in this study.

It is well known that the IJV cross‐sectional area and the cerebral venous flow are affected by change in central venous pressure in supine position (Gisolf et al. 2004). However, central venous pressure did not change during submaximal exercise in both upright and semi‐supine positions (Dawson et al. 2004; Trangmar et al. 2015). One reason for the decrease in the IJV diameter during moderate exercise is negative intrathoracic pressure by deep inspiration (Schaller 2004; Tsao et al. 2014). Moreover, the reduction in IJV cross‐sectional area might be due to a lower IJV intramural venous pressure associated with less venous blood volume, and less blood volume in the IJV may originate from ICA BF decrements at moderate exercise. Other possible explanations may involve neurogenic regulation of the venous tone to sympathoexcitation during exercise. The role of adrenergic innervation in venous tone regulation has been reported (Auer and Johansson 1981; Edvinsson et al. 1983; Nakakita et al. 1983; Itakura et al. 1984). Importantly, some humans studies reported indicate that the cerebral veins may possess an active rather than passive regulation of cerebral venous tone under some situations (Stolz et al. 2009, 2010; Ivanov et al. 2013). In contrast with the IJV, the VV was not constricted throughout the exercise (Fig. 1). Their different susceptibility to constriction is associated with the regional difference in flow resistance, sympathetic cerebrovascular control, and anatomical factors between the IJV and VV (Valdueza et al. 2000; Gisolf et al. 2004; Ogoh et al. 2016).

This study has several limitations. First, we were unable to simultaneously measure the BF in the cerebral arteries and veins during exercise. The time lag in BF measurements could certainly have an effect on the results of this study. However, based on the reproducibility of the BF results through repeated BF measurements during moderate exercise, we believe that this time lag only has a small effect. Second, IJV is a low‐pressure and compressible vessel. It is considered that decrease in IJV diameter during exercise is simply an artifact of IJV compression with the probe. However, in such case, the subcutaneous fat and muscle would be expected to be simultaneously compressed in addition to the vessel itself. As shown in Figure 1, such compression did not occur in this study. Finally, the sum of ICA and VA BF at rest and during exercise are not quantitatively matched to the sum of IJV and VV BF because we measured the right IJV. Unlike cerebral arteries, which supply BF to the cerebral hemisphere ipsilateral to each ICA, each IJV usually affects either deep cerebral venous system drainage (left IJV) or superficial cerebral venous drainage (right IJV) (Himwich et al. 1947; Suzuki et al. 2001; Chung et al. 2010). The CBF in cortical area is far higher than in the basal brain structures (Himwich et al. 1947; Suzuki et al. 2001).

In conclusion, we propose that the cerebral venous outflow was modified by dynamic supine exercise, and there is the coupling between arterial and venous BF regulation in both anterior and posterior cerebral circulation. It remains unclear whether changes in cerebral venous outflow influence on the regulation of CBF during supine exercise.

## Conflict of Interest

The authors declare no conflict of interest.
